# Evaluation of Anti-Adhesion Characteristics of Diamond-Like Carbon Film by Combining Friction and Wear Test with Step Loading and Weibull Analysis

**DOI:** 10.3390/ma14112746

**Published:** 2021-05-22

**Authors:** Hiroki Mano, Tsuguyori Ohana

**Affiliations:** Advanced Manufacturing Research Institute, National Institute of Advanced Industrial Science and Technology (AIST), 1-2-1 Namiki, Tsukuba, Ibaraki 305-8564, Japan; t.ohana@aist.go.jp

**Keywords:** diamond-like carbon, friction, wear, delamination, adhesion, high-frequency, linear-oscillation tribometer, Weibull distribution

## Abstract

Anti-adhesion characteristics are important requirements for diamond-like carbon (DLC) films. The failure load corresponding to the anti-adhesion capacity varies greatly on three types of DLC film (hydrogen-free amorphous carbon film (a-C), hydrogenated amorphous carbon film (a-C:H), and tetrahedral hydrogen-free amorphous carbon film (ta-C)) in the friction and wear test with step loading using a high-frequency, linear-oscillation tribometer. Therefore, a new method that estimates a representative value of the failure load was developed in this study by performing a statistical analysis based on the Weibull distribution based on the assumption that the mechanism of delamination of a DLC film obeys the weakest link model. The failure load at the cumulative failure probabilities of 10% and 50% increased in the order ta-C < a-C:H < a-C and ta-C < a-C < a-C:H, respectively. The variation of the failure load, represented by the Weibull slope, was minimum on ta-C and maximum on a-C:H. The rank of the anti-adhesion capacity of each DLC film with respect to the load obtained by a constant load test agreed with the rank of the failure load on each DLC film at the cumulative failure probability of 10% obtained by Weibull analysis. It was found to be possible to evaluate the anti-adhesion capacity of a DLC film under more practical conditions by combining the step loading test and Weibull analysis.

## 1. Introduction

DLC films are excellent tribo-coatings with low friction and high wear resistance [1,2]. Various analyses have been carried out on the tribological properties of DLC films over many years by both experiment and simulation [3,4]. Although DLC films have been applied to various fields, delamination is a serious problem, particularly in medical applications [5]. Many unclear points about the mechanism of delamination remain. To clarify the mechanism, as an example, results confirming the effect of residual stress due to surface roughness on the adhesion of the film obtained by numerical simulation and results evaluating mechanical properties, including the adhesion of the film and corrosion resistance, obtained by various test methods have been reported [6,7]. In addition, with the increased range of combinations of substrates and hard thin films, numerous efforts have been made to improve adhesion and suppress the occurrence of delamination by optimizing the substrate material, the film-forming conditions, and the intermediate layer [8,9,10,11].

The general evaluation method for the anti-adhesion characteristics of hard thin films is the scratch test specified in ISO 20502 [12]. Recently, various failure modes caused by changing the curvature of the indenter of the scratch test have been reported [13,14]. The Rockwell indentation test is also a typical evaluation method for the anti-adhesion characteristics of hard thin films specified in ISO 26443 [15]. As an example, for DLC films with or without an intermediate layer formed on titanium, the results of predicting the interface at which damage occurs from the observation of the cross-section of the Rockwell indentation and the calculation of stress, and the results of changing the bias voltage in small increments on the state of the dent have been reported [16,17]. Although these methods are useful for the quality control of films, such large-scale plastic deformation is unexpected under the stress conditions of practical application.

Friction and wear characteristics are important under stress conditions close to practical use, and in recent years, evaluation methods of these characteristics have been standardized as ISO 18535 [18]. On the other hand, the correlation between the results of the friction and wear test, and the results of the above-mentioned scratch test, have been not cleared for DLC films [19,20,21,22]. Furthermore, the fatigue resistance of DLC films is also important for their practical use, and an evaluation method based on repeated loading has been proposed [23]. The evaluation method for the anti-adhesion characteristics of DLC films combining repeated sliding and load increases includes every factor of the scratch test, friction and wear test, and fatigue test. For example, an evaluation method combining one-way rotation sliding with a gradual increase in load has been developed [18]. However, there are few reports on reciprocation, which is a typical motion form similar to one-way rotation [24]. Although various methods have been proposed for evaluating the anti-adhesion characteristics of DLC films, as described above, no established method has been found under conditions combining repeated sliding and repeated stress.

We are developing a novel evaluation method for the anti-adhesion characteristics of DLC films that combines reciprocating sliding and step loading using a high-frequency, linear-oscillation tribometer where its data reliability has been verified with multiple international round-robin tests [25]. As a result, it was clarified that the characteristics could be evaluated by measuring the load at which the DLC film was delaminated [26]. In this research, there are two types of processes in which delamination occurs, and it was clarified that the form of delamination depends on the presence or absence of embedded wear debris. However, it was suggested that the simple average value of the failure load was insufficient to represent the anti-adhesion characteristics of DLC films because the wear debris was embedded accidentally and the measured failure load varied greatly. Therefore, a statistical analysis based on the Weibull distribution was performed on failure load data measured using the method previously mentioned in this study. The Weibull distribution is one of the probability functions of failure [27] and is often used for the life evaluation of rolling bearings. Since the Weibull distribution can be applied to various phenomena, studies applying Weibull analysis to the life evaluation of molybdenum disulfide films measured by a reciprocating sliding tester [28], to evaluating the dispersion of wear loss and friction coefficient measured in a round-robin test [29], and to evaluating the seizure load measured by a combination of a reciprocating sliding test and a gradual increase in load [30] have been reported. In Weibull analysis, it is possible to quantitatively represent a phenomenon with a large variation by calculating the Weibull slope and the cumulative failure probability. This paper verified the basic feasibility of a new method that estimates the representative value of the failure load by combining the friction and wear test with step loading and Weibull analysis.

## 2. Experimental Procedure

### 2.1. Friction and Wear Tests with Step Loading

**Figure 1:**
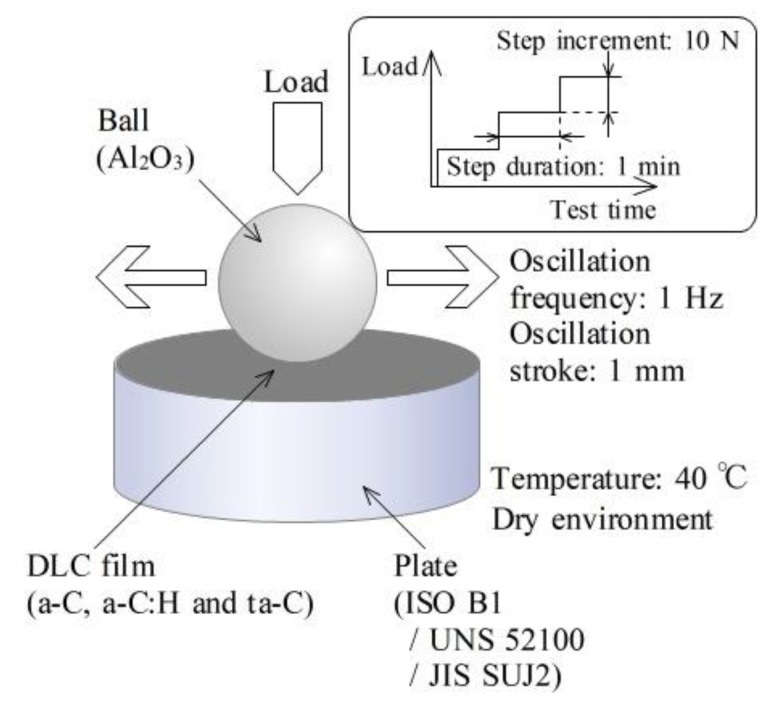
Experimental setup.

Figure 1 shows a schematic of the high-frequency, linear-oscillation tribometer (SRV model 3, Optimol Instruments Prüftechnik, Munich, Germany) used to evaluate the anti-adhesion characteristics of DLC films. A ball was pressed and rubbed against a plate. The friction coefficient was calculated based on force signals obtained from load cells installed directly under the stage of the plate and attached to the loading system of the ball. The operating conditions of the tribometer were a step loading, an oscillation frequency of 1 Hz, an oscillation stroke (double amplitude) of 1 mm, and a plate temperature of 40 °C in a dry environment (without lubricant oil). Because the atmospheric conditions affect the friction and wear characteristics of DLC films as reported in a previous study, a dry environment with a relative humidity of 34–49% was adopted to simplify the evaluation conditions. The normal load was increased in increments of 10 N every 1 min.

Three types of DLC film (hydrogen-free amorphous carbon film (a-C), hydrogenated amorphous carbon film (a-C:H), and tetrahedral hydrogen-free amorphous carbon film (ta-C) [31]) were deposited on a bearing-steel test plate (ISO B1/UNS 52100/JIS SUJ2 [32,33,34]) with a hardness of HV659 and a surface roughness of Ra 0.004 µm. The specifications of each DLC film are shown in Table 1. a-C, a-C:H, and ta-C were fabricated by unbalanced magnetron sputtering in a closed magnetic field, using ionized deposition and a filtered arc, respectively. a-C and a-C:H included an intermediate layer. The hardness and Young’s modulus of the film were measured using a nano indenter (Nano Indenter XP, MTS Systems Corporation, Oak Redge, TN, U.S.A.). The measurement method was the continuous stiffness measurement with an indentation depth of 500 nm, and the hardness at a depth of about 10% of the film thickness from the surface was defined as the hardness of the film. The surface roughness was measured using a stylus type surface profiler (Surfcom 1500SD, Tokyo Seimitsu Corporation, Tokyo, Japan) under the conditions of a measurement length of 0.4 mm and a radius of curvature of the tip of the stylus of 2 µm. All tests were executed on the same test plate of each DLC film. Al_2_O_3_ balls with a diameter of 9.525 mm used as rolling bearings were employed as test balls.

The conditions for test termination were the friction coefficient increasing by more than 0.05 from its value in the stable state and the oscillation stroke decreasing by more than 0.1 mm from its value in the stable state. The load when the test was terminated was evaluated as the failure load, which was considered to be the anti-adhesion capacity of the DLC film.

The tests were performed eight times on a-C, twelve times on a-C:H, and five times on ta-C to confirm repeatability. The wear scars were observed using an optical microscope and their cross-sections were measured using a surface profiler.

### 2.2. Weibull Analysis

A phenomenon that obeys the weakest link model can be analyzed with a Weibull distribution expressed by the following Equation (1) [27,35,36]:

(1) $$F\left(x\right)=1-\exp\left[-{\left(\frac{x}{\eta }\right)}^{m}\right]$$

here *F*(*x*) is the cumulative failure probability, *x* is the variant, *η* is the scale parameter, and *m* is the Weibull slope. When the natural logarithm of both sides of Equation (1) is taken twice, the following equation is obtained:

(2) $$\mathrm{lnln}\left(\frac{1}{1-F\left(x\right)}\right)=m\ln x+m\ln\frac{1}{\eta }.\;$$

The linearity of Equation (2) is reflected in the plot in the Weibull probability paper. In general, the Weibull distribution can be applied to the initial failure of the decreasing failure rate type, the contingency failure of the constant failure rate type, and the wear failure of the increasing failure rate type, depending on the size of the shape parameter. The Weibull distribution fits well with the distribution of the life and strength of metals and ceramics [37,38]. This suggests that regardless of the form of delamination occurring in the DLC film, it can be uniformly evaluated by Weibull analysis.

### 2.3. Friction and Wear Tests under Constant Load

A constant load test was carried out under loads of 50 N, 100 N, 150 N, and 200 N using the same tribometer as in the step loading test. The tribometer was operated under a constant load with an oscillation frequency of 1 Hz, an oscillation stroke (double amplitude) of 1 mm, a plate temperature of 40 °C, and a test duration of 1 h in a dry environment with a relative humidity of 34–49%.

The test specimens were identical to those in the step loading test. The occurrence of delamination was evaluated based on the trends of the friction coefficient and oscillation stroke during the test and the wear scar appearance after the test.

## 3. Results

### 3.1. Friction and Wear Tests with Step Loading

**Figure 2:**
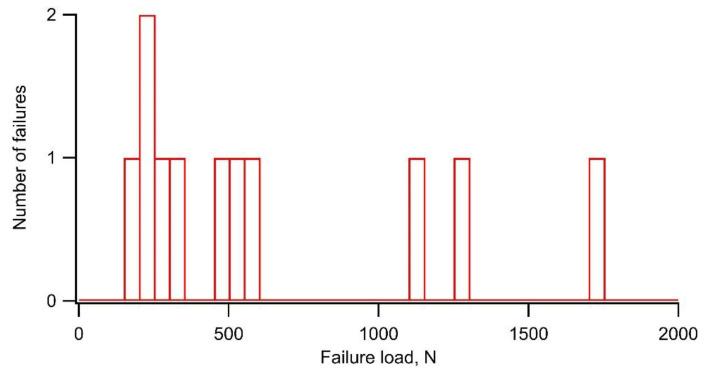
Distribution of the number of failures with respect to failure load on a-C:H films.

The results of the failure load, corresponding to the anti-adhesion capacity of the DLC film, in the eight tests on a-C, the twelve tests on a-C:H, and the five tests on ta-C are summarized in Table 2. The failure load was dispersed widely and varied from 210 N to 700 N on a-C, from 190 N to 1730 N on a-C:H, and from 60 N to 120 N on ta-C. Figure 2 shows the distribution of the number of failures with respect to the failure load for a-C:H. Although the average value of the failure load was 630 N, the distribution of the number of failures was not bilaterally symmetrical about the average value. Therefore, the average value is not suitable as a representative value of the failure load. The Weibull distribution fitting is considered suitable for the shape of this distribution.

**Figure 3:**
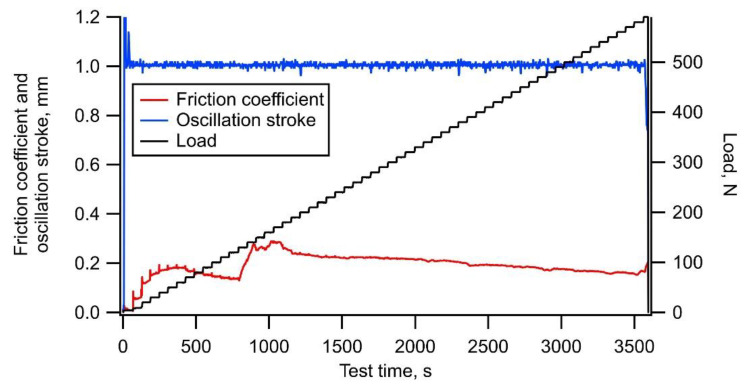
Typical example of trends of friction coefficient, oscillation stroke, and load during test using a-C film.

**Figure 4:**
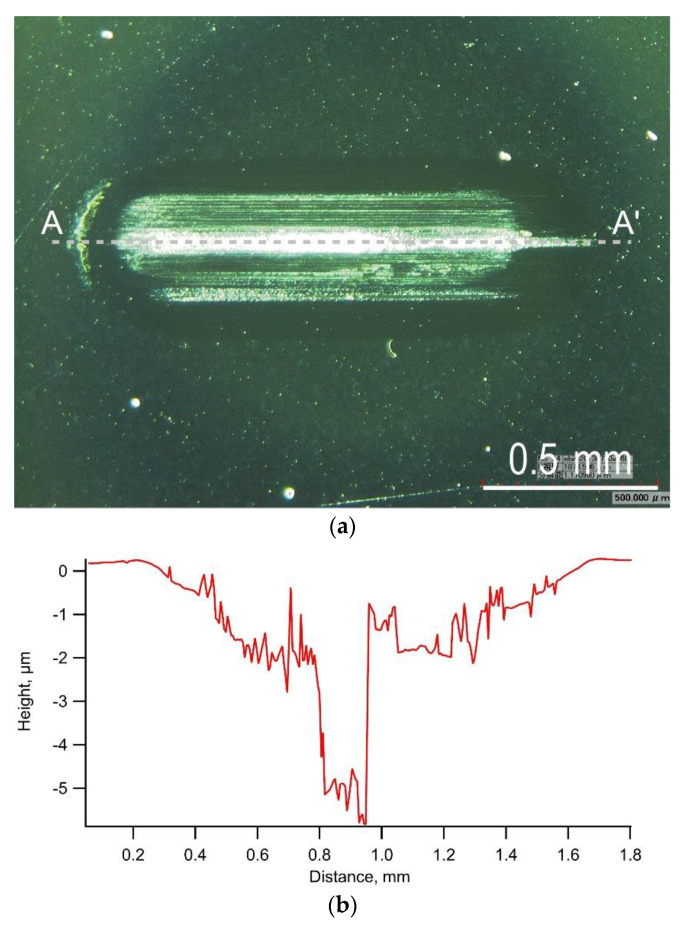
Typical example of the appearance of wear scar and its cross-section shape observed in the test using a-C film: (**a**) Appearance of wear scar on test plate, (**b**) Cross-section shape along A-A’.

Figure 3 shows the typical trends of the friction coefficient, oscillation stroke, and load during a test on a-C. The friction coefficient increased and gradually decreased at the start of the loading, then increased and gradually decreased again during the test, and finally increased rapidly with delamination occurring at a load of 590 N. The oscillation stroke decreased rapidly when the delamination occurred. The friction coefficient was considered to have fluctuated due to the embedment of a part of wear debris generated in large quantities [26]. Figure 4a,b shows the appearance of the wear scar on the test plate and its cross-section shape, respectively. Many deep scratch marks due to severe wear and local delamination with a depth greater than the film thickness of 1.0 µm were observed inside the wear scar.

**Figure 5:**
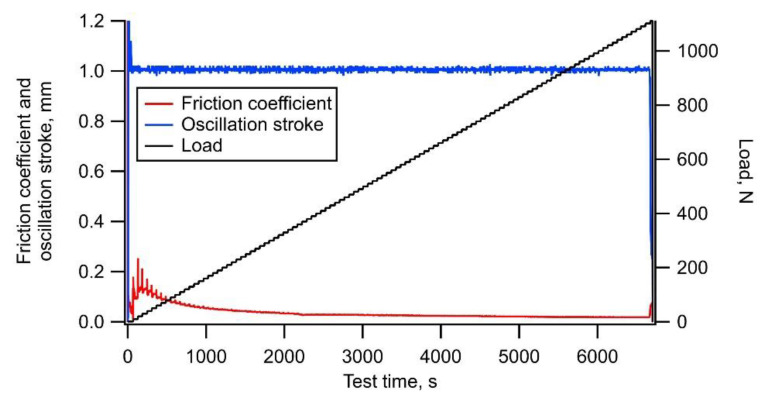
Typical example of trends of friction coefficient, oscillation stroke, and load during test using a-C:H film.

**Figure 6:**
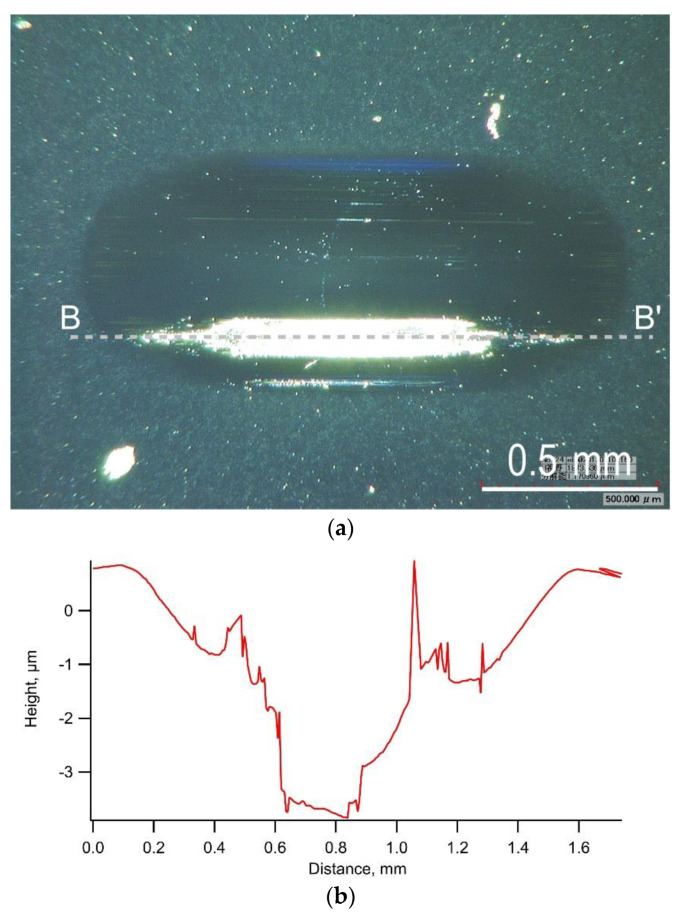
Typical example of the appearance of wear scar and its cross-section shape observed in the test using a-C:H film: (**a**) Appearance of wear scar on test plate, (**b**) Cross-section shape along B-B’.

Figure 5 shows the typical trends of the friction coefficient, oscillation stroke, and load during a test on a-C:H. Although the friction coefficient increased at the start of the loading, it gradually decreased, and finally increased rapidly with delamination occurring at a load of 1110 N. The oscillation stroke decreased rapidly when the delamination occurred, similar to a-C as shown in Figure 3. The friction coefficient was considered to have transitioned smoothly as mild wear progressed. Figure 6a,b show the appearance of the wear scar on the test plate and its cross-section shape, respectively. Some slight scratch marks due to the mild wear and large delamination with a depth greater than the film thickness of 0.8 µm were observed inside the wear scar.

**Figure 7:**
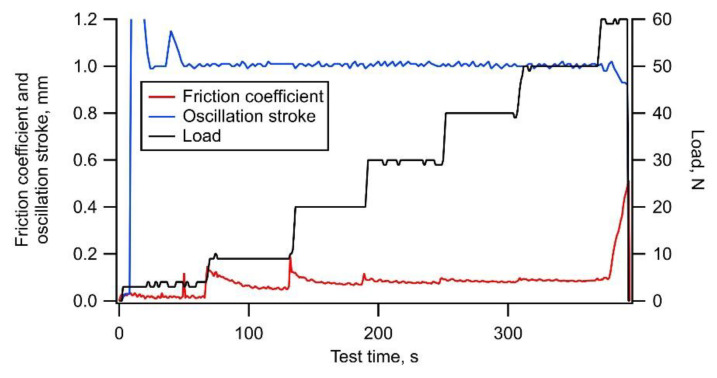
Typical example of trends of friction coefficient, oscillation stroke, and load during test using ta-C film.

**Figure 8:**
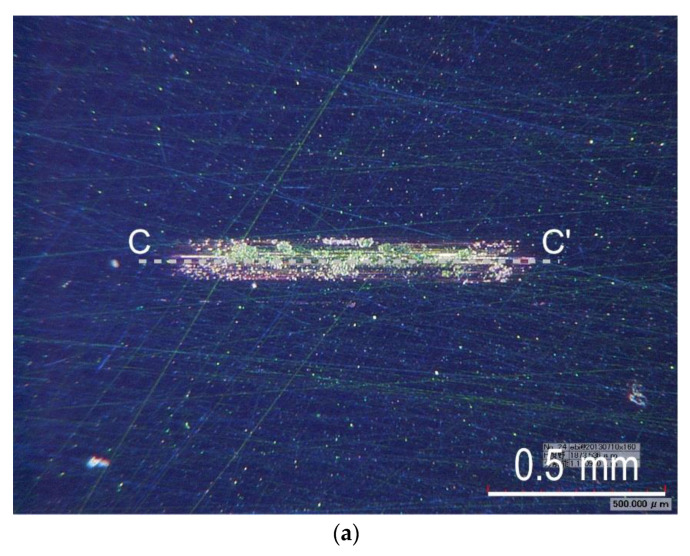

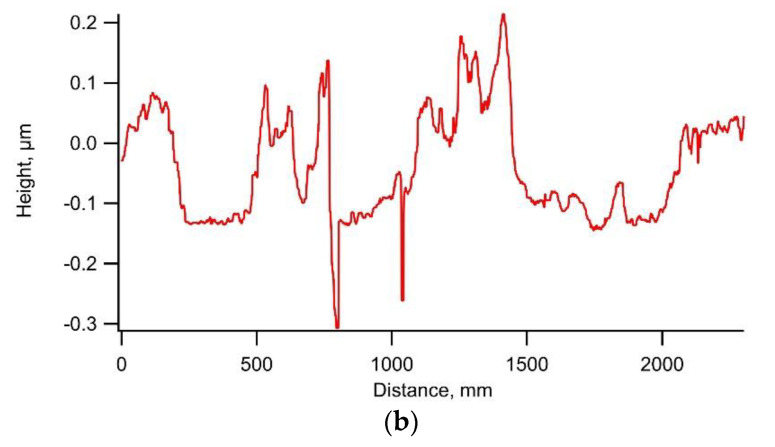
Typical example of the appearance of wear scar and its cross-section shape observed in the test using ta-C film: (**a**) Appearance of wear scar on test plate, (**b**) Cross-section shape along C-C’.

For ta-C, approximately half of the test showed the same trend as in Figure 7. The friction coefficient for these tests showed a substantially constant value from the start of loading and finally increased rapidly with delamination occurring at a load of 60 N. The friction coefficient was considered to have turned to a rapid increase in a short period due to the accumulation of wear debris and the brittle fracture of the film. Figure 8a,b show the appearance of the wear scar on the test plate and its cross-section shape, respectively. Convexity, which was caused by the accumulation of wear debris, and local small delamination with a depth equal to 0.1 µm of the film thickness were observed inside the wear scar.

**Figure 9:**
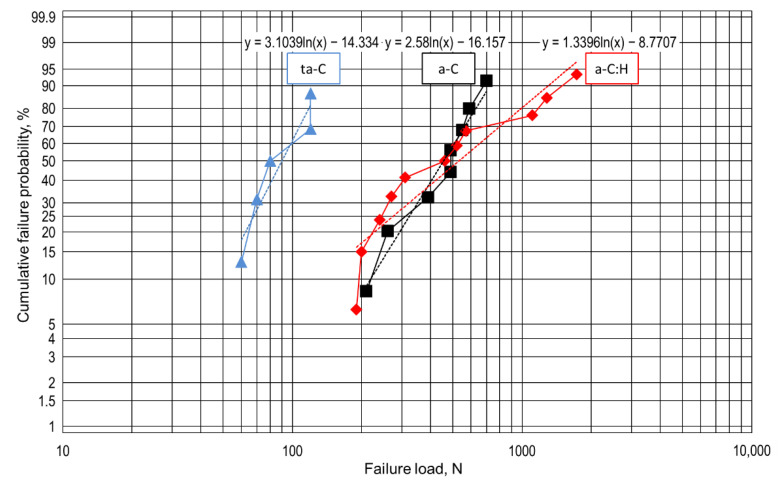
Weibull plot of failure load on DLC films (a-C, a-C:H, and ta-C).

The failure load on each DLC film varied greatly even under the same experimental conditions: therefore, assuming the mechanism of delamination obeyed the weakest link model, and statistical analysis was performed based on the Weibull distribution. The values in Table 2 were plotted on Weibull probability paper as shown in Figure 9. When delamination did not occur, the load on the test was treated as a censored data [39]. The Weibull slope calculated from the regression line of the group of data and the failure load at the cumulative failure probabilities of 10% and 50% on each DLC film is summarized in Table 3. The parameters of the regression line were estimated by the least squares method. The failure load at the cumulative failure probabilities of 10% and 50% increased in the order ta-C < a-C:H < a-C and ta-C < a-C < a-C:H, respectively. The variation of the failure load represented by the Weibull slope was minimum on ta-C and maximum on a-C:H. The correlation coefficients of a-C, a-C:H, and ta-C in this Weibull analysis were 0.980, 0.928, and 0.944, respectively.

### 3.2. Friction and Wear Tests under Constant Load

The validity of the proposed method for evaluating the anti-adhesion capacity of DLC films by combining the friction and wear test with step loading and Weibull analysis was verified using the friction and wear test under multiple constant loads. The results are summarized in Table 4. The load conditions were 50 N, 100 N, 150 N, and 200 N. ta-C failed to complete the 1 h test under 50 N and 100 N. One of the a-C:H tests completed the 1 h test under 100 N, but the other failed to complete the test under the same load, and a-C:H failed to complete the test under 150 N. a-C completed the 1 h test under all load conditions. Here, 50 N corresponds to the failure load at the cumulative failure probability of 10% on ta-C (49.1 N), 150 N corresponds to the failure load at the cumulative failure probability of 10% on a-C:H (130.0 N), and 200 N corresponds to the failure load at the cumulative failure probability of 10% on a-C (219.2 N). Delamination occurred at loads of both 50 N and 100 N on ta-C. For a-C:H, delamination also occurred at a load of 150 N, but there was a case where delamination occurred with a load of 100 N and a case where it did not occur. No delamination occurred at loads of 200 N, 150 N, and 100 N on a-C. Accordingly, the rank of the anti-adhesion characteristics was ta-C < a-C:H < a-C in the constant load test, which was the same order as for the failure load at the cumulative failure probability of 10% measured in the step loading test.

## 4. Discussion

The main results obtained by the Weibull analysis are summarized below:

- The representative values of the failure load are high on the thicker a-C and a-C:H films, whereas the representative value of the failure load is relatively low on the thinner ta-C film.
- The variation of the failure load on a-C:H is larger than that on a-C.

The rank of the anti-adhesion capacity of each DLC film with respect to the load obtained by the constant load test agreed with the rank of the failure load on each DLC film at the cumulative failure probability of 10% obtained by Weibull analysis. Therefore, the validity of the proposed method for evaluating the anti-adhesion capacity of the DLC film by combining the friction and wear test with step loading and Weibull analysis has been shown. By applying Weibull analysis, it is possible to evaluate the resistance of a DLC film to repeated sliding and repeated stress. From the viewpoint of evaluation at loads closer to those in practical use, new knowledge can be obtained by combining the step loading test and Weibull analysis. Under conditions where large deformation does not occur, the influence of wear and embedded wear debris is not negligible. It has already been reported that the Weibull distribution can be applied to the adhesion stress of thin films measured by pull tests [40] and the critical load for delamination measured in scratch tests [41,42,43], and a new possible application of the Weibull distribution has been clarified in this paper. In Figure 9, the Weibull plot corresponding to a-C:H shows that the slope is different between high failure loads and low failure loads, and as a result, the plot seems to contain a kink. When data of different populations are combined into one Weibull plot, the plot bends [28,29]. Therefore, since there is a possibility that data of different delamination forms overlap on a-C:H, it is considered that the correlation coefficient became the minimum. In the case of a-C, wear is the main cause of delamination, so there is less variation of the failure load. On the other hand, since a-C:H has two causes of wear and excessive load, the variation is relatively large. For ta-C, the internal stress was not measured because a thick film could not be formed on a Si wafer, but high internal stress is considered to be one of the reasons for the low failure load. In addition, the ta-C film was also considered to be disadvantageous from the viewpoint of the ability to follow the deformation of the substrate because it was harder than the other films and its Young’s modulus was also larger.

It is considered that one of the causes of the large variation in the failure load even with the same test film is the wear behavior during the running-in process. The wear behavior is affected not only by the heterogeneity of the film quality, including adhesion but also by small differences in the sliding and environmental conditions. As the results of general friction and wear tests also vary, various factors act in a complicated manner on the friction and wear characteristics, and it is a future task to clarify the cause of the variation in the failure load.

The friction coefficient may have an effect on delamination, but the friction coefficient on DLC film is generally low and the frictional resistance is presumed to be not so large, so the magnitude of the friction coefficient is considered to not be the dominant factor of the delamination. The differences in the values of failure load and Weibull slope for each specimen at cumulative failure probabilities of 10% and 50% are due to the combined effects of various factors. In practice, the overall performance under the influence of these factors is important, and this paper proposes a method to quantitatively express the performance. The mutual comparable information on the anti-adhesion capacity of the film can be obtained by performing evaluations with the same conditions using this method.

In this study, it was shown that the anti-adhesion characteristics of DLC films can be evaluated in an environment accompanying sliding, different from the scratch test and the Rockwell indentation test. Moreover, it was found that accurate evaluation is difficult unless the form of delamination is considered. The effect of film thickness will be reported while considering the form of delamination in our next paper.

## 5. Conclusions

In the friction and wear test with step loading using a high-frequency, linear-oscillation tribometer, the failure load corresponding to the anti-adhesion capacity was measured for three types of DLC film (a-C, a-C:H, and ta-C). Since the failure load on each DLC film varied greatly, it was assumed that the mechanism of delamination of the DLC film obeyed the weakest link model, and a new method of estimating a representative value of the failure load was developed by performing a statistical analysis based on the Weibull distribution. The main results are as follows:

(1) The failure load at the cumulative failure probabilities of 10% and 50% increase in the order ta-C < a-C:H < a-C and ta-C < a-C < a-C:H, respectively. The variation of the failure load, represented by the Weibull slope, was minimum on ta-C and maximum on a-C:H.
(2) The rank of the anti-adhesion capacity of each DLC film with respect to the load obtained by a constant load test agreed with the rank of the failure load on each DLC film at the cumulative failure probability of 10% obtained by Weibull analysis.
(3) It was found to be possible to evaluate the anti-adhesion capacity of a DLC film under more practical conditions by combining the step loading test and Weibull analysis, i.e., a different viewpoint from the scratch test and the Rockwell indentation test.

Although some tests showed high failure loads, there is a possibility that the adhesiveness was improved by the penetration effect of the DLC film accompanying the deformation of the substrate. In addition, the sliding conditions may have affected the failure load, and further study is necessary.

## Figures and Tables

**Table 1:** Specifications of DLC films (a-C, a-C:H, and ta-C).

Film Property	a-C	a-C:H	ta-C
Film thickness, µm	1.0	0.8	0.1
Hardness, GPa	15	23	52
Young’s modulus, GPa	165	193	446
Surface roughness (Ra), µm	<0.008	<0.008	<0.008
Surface roughness (Rz), µm	≈0.040	≈0.040	≈0.048

**Table 2:** Test results of failure load on DLC films (a-C, a-C:H, and ta-C). Values are in N.

Test No.	a-C	a-C:H	ta-C
1	260	270	120
2	210	1280	120
3	490	1110	80
4	390	460	60
5	590	90 *	70
6	550	200	-
7	490	190	-
8	700	1730	-
9	-	240	-
10	-	520	-
11	-	310	-
12	-	570	-

* Undamaged, treated as abortive data.

**Table 3:** Weibull slope and failure load at cumulative failure probabilities of 10% and 50% on DLC films (a-C, a-C:H, and ta-C).

Film Type	Weibull Slope	Failure Load, N		Correlation Coefficient
		Cumulative Failure Probability of 10%	Cumulative Failure Probability of 50%	
a-C	2.58	219.2	455.0	0.980
a-C:H	1.34	130.0	530.0	0.928
ta-C	3.10	49.1	90.0	0.944

**Table 4:** Test results of DLC films (a-C, a-C:H, and ta-C) under multiple constant loads.

Film Type		50 N	100 N	150 N	200 N
ta-C	1	NG (1 min)	NG (1 min)	–	–
	2	–	–	–	–
a-C:H	1	–	Good	NG (2 min)	–
	2	–	NG (3 min)	–	–
a-C	1	–	Good	Good	Good
	2	–	–	Good	–

Good: No delamination, NG: Delamination, ( ): Test time at which delamination is considered to have occurred, –: No test.

## Data Availability

Data sharing is not applicable.
